# Supplementary material for: Applying Super-Resolution and Tomography Concepts to Identify Receptive Field Subunits in the Retina
Source: PLoS Comput Biol. 2024 Sep 3;20(9):e1012370. doi: 10.1371/journal.pcbi.1012370 (PMC11398665; doi:10.1371/journal.pcbi.1012370)
Supplement: Attachment — Submitted filename: point_by_point_reply.pdf [file pcbi.1012370.s001.pdf]

We would like to thank the reviewer for their effort and for the helpful, constructive feedback. In the revised manuscript, we have addressed the points raised by the reviewer as detailed in the responses below. In particular, we have added a comprehensive analysis of how measurement time and accuracy trade off with the underlying number of subunits, and a comparison with spike-triggered clustering (new Figure 4). Furthermore, we have extended our analysis of the robustness of the method to model variations to more structural changes like superimposed subunit layouts and spatial nonlinearities within subunits (new Figure 6). We believe that these changes have helped improve the manuscript by underscoring the central results and clarifying some of the explanations, and we thank the reviewer for their suggestions.

Time required

One of the arguments made in favor of the new approach is that it requires less time to identify subunits. It is not clear from the results in the paper whether this is the case, and if it is how much of a benefit the approach represents. In the Shah et al. (2020) paper, subunits are identified based on white noise stimulation in ~20 min. The approach presented here would appear to require a similar amount of time. But that is a very qualitative comparison - and one or the other approach may be more efficient. The paper would benefit substantially from a direct comparison of a noise-based approach (e.g. the non-negative matrix factorization approaches used by the Gollisch group in the past or the Shah et al. approach) and the current approach.

We agree that the improvements over existing methods were not well delineated. Since spike-triggered non-negative matrix factorization (Liu et al., 2017) is reported to typically require a minimum of 1-2 hours of data, which is substantially more than our method, we have focused on spike-triggered clustering (Shah et al., 2020). As correctly pointed out, spike-triggered clustering can recover spatial filters with less than 30 minutes of white noise data, though longer recordings with more finely-grained spatial structure lead to more and smaller spatial filters. We have thus applied spike-triggered clustering to simulated responses with 30 minutes of white noise, where we have chosen stimulation parameters (e.g. frame rate of 60 Hz) to be as close to the original report as possible (Fig 4J). Even though we supplied spike-triggered clustering with the ground truth number of subunits and the ground truth temporal filter, it only recovers a few aggregates of the underlying subunits from our simulated data. This is in line with the observations in the original paper, where, even in their own simulations of short white noise stimulation (Fig S1B in Shah et al. (2020)), the authors noted spatial filters to be aggregates of subunits/bipolar cells. Only with much longer and finer white noise stimulation does spike-triggered clustering recover more underlying subunits, but still many filters are aggregates of subunits (with long fine white noise stimulation, Shah et al. (2020) did recover simulated subunits (Fig S1C), but their direct stimulation of simulated photoreceptors drastically reduced the dimensionality of the problem compared to our case). In summary, while spike-triggered clustering can recover coarse spatial filters with a similarly short recording duration, it only identifies the actual underlying subunits with substantially more data than our method requires. We have added this information to the manuscript (Fig 4J and lines 353 ff.). We refrained from calculating F-scores for spike-triggered clustering, since our goal is not to diminish the usefulness of that method, and due to our lack of experience with it we cannot guarantee that we have applied it in the best possible way. Additionally, we investigated the measurement time that our STR method requires in more detail, as described below ("Extend analysis of accuracy as number of subunits increases"), which further strengthens the comparison of STR with spike-triggered clustering.

Overlapping subunits

Many RGC types receive input from several bipolar types (as mentioned in the Discussion). That might be expected to create overlapping subunits. The Discussion mentions that these may be hard to resolve, but it would be nice to see an analysis of this situation and a discussion based on this analysis of its importance.

Thank you for the feedback. We have taken the opportunity to test three different cases of superimposed subunit layouts: two layouts with the same basic properties (subunit number and sizes), two layouts with differently sized subunits, and two layouts with opposing response polarity. We find that, while differently sized subunits can be independently reconstructed quite well, the cases of the

same properties or opposing response polarities pose a bigger challenge. Note that we have assumed the two layouts to contribute with equal weights to the model's responses, which is the most difficult scenario, and many ganglion cell types likely receive principal, though not exclusive input from one type of bipolar cells. We have added new figures (Fig 6A-C) and explanations to the results (lines 458 ff.) and extended the discussion accordingly (lines 810 ff.).

Robustness to multiple sources of subunits

Work from some of the same authors shows nicely that subunits are already present in the bipolar input signals. How robust is the approach to having several sequential sources of subunits? An analysis of this possibility and how well (or not well) it is handled would enhance the paper.

Indeed, our lab has previously demonstrated that some bipolar cells in the salamander retina already integrate visual stimuli spatially nonlinear (Schreyer and Gollisch, 2021). We have now tested this scenario by adding a layer of simulated photoreceptors that nonlinearly send signals to the bipolar-cell subunits, extending our model to an LNLNLN model. Naturally, the impact of these simulated photoreceptors depends on their nonlinearity. If the photoreceptor nonlinearity is fully rectifying (same as the subunit nonlinearity), the bipolar-cell subunits are mathematically irrelevant in our simulations and the photoreceptors would represent the only relevant subunits and could be identified with our method. We decided to focus on a moderate photoreceptor nonlinearity (piecewise linear with slope of 0.5 and unity for negative and positive inputs, respectively), which presumably is also the more realistic scenario compared to a fully rectified photoreceptor output. Interestingly, this only has a marginal impact on the recovery of the bipolar-cell subunits (Fig 6D and lines 500 ff. in the updated manuscript). The photoreceptor layout could in principle also be reconstructed when very fine stripes and noise-free measurements are considered, but we decided to only mention this in passing in the manuscript since the responses to the fine stripes are likely too weak with more realistic spike generation.

Experimental validation

The experimental validation of the approach is an important addition but is less complete than the remainder of the paper. At present, it consists of a few example cells but no real analysis of cell populations. Is the number of subunits identified across ganglion cells of the same type consistent? What about the subunit sizes? The number of identified subunits (e.g. four in the case of the On parasol shown in Fig. 6) seems quite low; can you compare that to expectations from anatomy and comment on any discrepancy? On the technical side, is the 375 micron separation of the stripes sufficient to avoid surround activation?

Thank you for the feedback. As suggested, we have extended the analysis of the experimental data by adding population analysis, focusing on subunit number and sizes (Fig 8H, I and lines 653 ff. in the updated manuscript). Off and On parasol ganglion cell reconstructions contained a median of 4 and 6.5 hotspots, respectively. This is indeed less than what might be expected from anatomy, but similar to previous functional estimates. The estimated size of subunits (approximated via the nearest neighbor distance of hotspots) of around 35 µm lies in the expected range. These analyses support the applicability of the STR method to experimental data and also highlight challenges for further improvement, in particular regarding the applied reconstruction algorithm, which may currently limit the detection of subunits with small weights. Future investigations will therefore both tackle alternatives to the filtered back-projection algorithm as well as new experiments that probe additional stimulus parameter settings and aim for higher quality data to go beyond the proof-of-principle analysis of the currently available recordings.

Future explorations of experimental parameter setting may also concern the separation between parallel stripes. However, the 375 µm separation used for the present data may be seen as a reasonable balance to minimize interactions between stripes and maximize recording efficiency. In particular, it was selected to ensure that receptive field centers (typically around 150 µm in diameter or less; see lines 559 ff.) are always stimulated by at most a single stripe. Nonetheless, neighboring stripes may hit the surround of a cell, but we mostly expect a generic influence on response strength with little influence on the structure extracted in the receptive field center. We have clarified these aspects in the manuscript (lines 570 ff.).

Nature of errors

Are the errors that the algorithm makes largely missing or mislocalizing subunits (or a combination of the two)?

We have now added this information to the manuscript (lines 272 ff.). Undetected subunits make up 54% of errors, spurious detections 31%, and mislocalizations (defined as a hotspot in the 1.5 σ but not 0.75 σ ellipse) 14% across layouts with our basic simulation settings. Note that in the F-score that we use for quantifying subunit detection, mislocalizations count twice – both as an undetected subunit and a spurious detection. Furthermore, the exact ratios of errors vary with the considered scenario, e.g., with the number of subunits, but the prevalence of undetected subunits is quite common. While this might suggest that hotspots should be detected more aggressively, we found that changing the hotspot detection threshold (currently at 0.3 of the global maximum) did not significantly impact performance.

Extend analysis of accuracy as number of subunits increases

Prior approaches to identify subunits often end up with fewer than expected based on anatomical considerations, suggesting that the functional subunits are in fact combinations of several anatomical subunits. This has long been puzzling. Figure 3 investigates this issue nicely. It would be helpful to extend that analysis to larger numbers of subunits (16 is probably close to a minimal number for most ganglion cell types, and many likely have quite a few more subunits). More exploration of how time and number of subunits trade off would also be helpful - e.g. if the number of subunits doubled, how much additional time would be required to compensate and recover equally accurate estimates?

Thank you for this suggestion. We have extended our investigations of reconstruction performance and optimal parameters to up to 30 subunits (Fig 4). Here, we also explore how performance increases with increasing measurement time and what measurement time is required to reach a certain performance. This reveals that the required measurement time grows approximately with the square of the number of subunits, but starts at a low baseline. In our simulations, only roughly ten minutes are sufficient to decently reconstruct layouts of ten subunits. As part of this analysis, we furthermore discuss how to systematically adjust reconstruction parameters, such as used for smoothing the sinograms, according to the expected number of subunits. Finally, we have also added an example showing that even large layouts of 50 subunits can in theory be reconstructed with STR. These results are shown in the new Figure 4 (A-I) and discussed in the text (lines 278 ff.).

Responses to bar offsets

Have you tried using responses to both the onset and the offset of the bars in your analysis (similar to looking at a frequency-doubled response to isolate the nonlinear component of a contrast-reversing grating)? It might decrease sensitivity to the RF itself and increase sensitivity to the subunits.

This is an interesting idea. We have checked both the on- as well as offset response for all cells in our recording, and as already reported in the previous version of the manuscript, offset responses under the dark-centered Ricker stripes can be used to extract receptive field substructure for On parasol cells. However, the unpreferred change at the Ricker stripe center (offset for Off cells, onset for On cells) does not reveal any hotspot structure in the receptive field center (cf. Fig 8E, bottom row). Moreover, for these conditions, responses seem to be mainly triggered by the sidebands falling onto and exciting the receptive field center and thus do not reflect the receptive field structure at the actual stripe center. We also do not observe responses equivalent to a frequency doubling; in our case, when the onset of a Ricker stripe with a given position and angle strongly activates a cell, the offset typically doesn't, and vice versa. Consequently, there doesn't appear to be a direct way to combine onset and offset responses to improve reconstructions. In the revised manuscript, we now discuss offset responses in more detail (lines 622 ff.).
